# A One Health Approach to Strengthening Antimicrobial Stewardship in Wakiso District, Uganda

**DOI:** 10.3390/antibiotics9110764

**Published:** 2020-10-31

**Authors:** David Musoke, Freddy Eric Kitutu, Lawrence Mugisha, Saba Amir, Claire Brandish, Deborah Ikhile, Henry Kajumbula, Ismail Musoke Kizito, Grace Biyinzika Lubega, Filimin Niyongabo, Bee Yean Ng, Jean O’Driscoll, Kate Russell-Hobbs, Jody Winter, Linda Gibson

**Affiliations:** 1 Department of Disease Control and Environmental Health, School of Public Health, College of Health Sciences, Makerere University, Kampala P.O. Box 7072, Uganda; gracelubega45@gmail.com (G.B.L.); filiminniyongabo@gmail.com (F.N.); 2 Department of Pharmacy, School of Health Sciences, College of Health Sciences, Makerere University, Kampala P.O. Box 7072, Uganda; kitutufred@gmail.com; 3 College of Veterinary Medicine, Animal Resources and Biosecurity (COVAB), Makerere University, Kampala P.O. Box 7062, Uganda; mugishalaw@gmail.com; 4 School of Animal, Rural and Environmental Sciences, Nottingham Trent University, Nottingham NG25 0QF, UK; saba.amir@ntu.ac.uk; 5 Buckinghamshire Healthcare NHS Trust, Aylesbury HP21 8AL, UK; claire.brandish@nhs.net (C.B.); beeyean.ng@nhs.net (B.Y.N.); jean.odriscoll1@nhs.net (J.O.); kate.russellhobbs@nhs.net (K.R.-H.); 6 Institute of Health and Allied Professions, School of Social Sciences, Nottingham Trent University, Nottingham NG1 4FQ, UK; deborah.ikhile@ntu.ac.uk (D.I.); linda.gibson@ntu.ac.uk (L.G.); 7 Department of Medical Microbiology, School of Biomedical Sciences, College of Health Sciences, Makerere University, Kampala P.O. Box 7072, Uganda; henrykajumbula427@gmail.com; 8 Entebbe Regional Referral Hospital, Entebbe P.O. Box 29, Uganda; ismailkizito11@gmail.com; 9 Department of Biosciences, School of Science and Technology, Nottingham Trent University, Nottingham NG11 8NS, UK; jody.winter@ntu.ac.uk

**Keywords:** antimicrobial resistance, antimicrobial stewardship, community health workers, health practitioners, infection prevention and control, multidisciplinary, one health, Uganda, UK

## Abstract

Antimicrobial stewardship (AMS), as one of the global strategies to promote responsible use of antimicrobials to prevent antimicrobial resistance (AMR), remains poor in many low-and middle-income countries (LMICs). We implemented a project aimed at strengthening AMS in Wakiso district, Uganda using a One Health approach. A total of 86 health practitioners (HPs), including animal health workers, and 227 community health workers (CHWs) participated in training workshops, and over 300 pupils from primary schools were sensitized on AMR, AMS, and infection prevention and control (IPC). We further established two multidisciplinary online communities of practice (CoPs) for health professionals and students, with a current membership of 321 and 162, respectively. In addition, a Medicine and Therapeutics Committee (MTC) was set up at Entebbe Regional Referral Hospital. The project evaluation, conducted three months after training, revealed that the majority of the HPs (92.2%) and CHWs (90.3%) reported enhanced practices, including improved hand washing (57.3% and 81.0%, respectively). In addition, 51.5% of the HPs reported a reduction in the quantity of unnecessary antibiotics given per patient. This project demonstrates that AMS interventions using a One Health approach can promote understanding of the prudent use of antimicrobials and improve practices at health facilities and in communities.

## 1. Introduction

Antimicrobial resistance (AMR) poses a global public health concern that relates to humans, animals, and the environment. Several factors contribute to the escalation of AMR, including inappropriate prescription, misuse and overuse of, and lack of effective stewardship of antimicrobials [1]. In 2015, the World Health Assembly endorsed a global action plan to tackle the worldwide problem of AMR [1]. This plan has at its core the use of a One Health multi-sectoral approach, and calls for collaboration and co-ordination globally and locally. In response, Uganda developed and released its 5-year AMR National Action Plan (NAP) in 2018, which sets out a framework of actions to address the undertakings across the country [2]. The NAP acknowledges limited awareness of AMR and data pertaining to antimicrobial use, rising rates of AMR in the country, and the comprehensive steps that need to be taken to contain and control this threat to global health [2,3,4].

One Health refers to a collaborative, co-ordinated, and multidisciplinary approach to ensure the health and wellbeing of humans, animals, and the environment across different spatial levels [5]. A One Health approach is necessary as AMR is an ecological challenge that is affected by the interrelations between humans, animals and the environment [5]. The implementation of interventions and actions of multiple actors towards the optimization of antimicrobial use is known as antimicrobial stewardship (AMS) [6]. Despite increasing evidence for the need for a multidisciplinary approach to tackle AMR, the use of One Health in addressing AMS challenges has been minimal. A recent systematic review showed that there is a dearth in the practice and implementation of AMS programs across Africa [7]. Whereas there is increasing evidence on challenges affecting AMS in Uganda, there is little literature on antimicrobial use in animals and its relationship to human health and the environment [8,9,10]. This therefore calls for more interventions to use a multidisciplinary approach to improve AMS across the country as stipulated in the NAP [2].

With support from the Commonwealth Partnership for Antimicrobial Stewardship (CwPAMS) scheme [11], an initiative of Commonwealth Pharmacists Association (CPA) and Tropical Health and Education Trust (THET) under the Fleming Fund of the UK Department of Health and Social Care (DHSC), our health partnership aimed to strengthen AMS in Wakiso district, Uganda. The focus of the project was on capacity building, multidisciplinary stakeholder engagement, and knowledge exchange using a One Health approach. The project drew on a multidisciplinary partnership and expertise from: the Schools of Social Sciences, Animal, Rural and Environmental, and Science and Technology at Nottingham Trent University (NTU); Buckinghamshire Healthcare NHS Trust (BHT); Colleges of Health Sciences, and Veterinary Medicine, Animal Resources and Biosecurity at Makerere University (Mak); and Entebbe Regional Referral Hospital (ERRH) in Uganda.

It is acknowledged that antimicrobials are used widely in both humans and animals, and are commonly present in the environment [12], hence the need for a broader One Health approach in addressing AMR. Although most AMS interventions have been health facility based, a large proportion of antimicrobials are used in the community, both as part of outpatient care [13] and integrated community case management (iCCM) for the treatment of childhood illnesses. In iCCM, community health workers (CHWs) are involved in the diagnosis of malaria, diarrhea, and pneumonia among children under five years of age. CHWs are the frontline health cadre at the community level in many low- and middle-income countries (LMICs), including Uganda, and so, they have a key role in ensuring proper use of antimicrobials in their communities. Therefore, this project was designed with interventions at both health facility and community levels to ensure wide reach and impact. It was also planned for knowledge to cascade from healthcare professionals into the wider communities through the CHWs. Specifically, the project aimed to: strengthen AMR awareness and upskill human and animal health practitioners (HPs) in AMS and infection prevention and control (IPC); utilize a training of trainers approach with the HPs and CHWs to improve community-wide awareness of AMR; establish communities of practices (CoPs) for sustainable engagement and resource sharing to support AMS; and facilitate knowledge exchange and sharing of best practice between Uganda and UK. In this paper, we describe the main activities and achievements of the project, including results from the evaluation of the HPs and CHWs who were involved in the training workshops.

## 2. Materials and Methods

### 2.1. Project Site and Setting

The 15-month project, as part of the CwPAMS scheme, was implemented in Wakiso district, central Uganda. Wakiso district has a total surface area of 2807.75 square kilometers, and a population of 2,007,700 people at an estimated growth rate of 4.1% [14]. ERRH, located in Entebbe municipality, Wakiso district, is the health facility where the main project interventions were implemented. ERRH has a 200-bed capacity, serving approximately 300 to 400 out-patients per day. Services offered at the hospital include but are not limited to: dental, pharmacy, peadiatrics, radiology, laboratory, maternity, maternal and child health, general surgery, internal medicine, and orthopedics. The hospital is led by a medical director, and approximately 10% of its staff are prescribers, including medical officers, dental surgeons, and clinical officers. ERRH serves a population of over 300,000, including the community in Entebbe municipality and neighboring areas, some being islands on Lake Victoria [14]. The community component of the project was implemented in Busiro South Health Sub District (HSD) in Wakiso district, which is comprised of three town councils (Kajjansi, Kasanje, and Katabi) and one sub county (Bussi). The HSD has a population of approximately 243,420 people. With a high number of households in Busiro South and the wider Wakiso district engaged in poultry and livestock farming, antimicrobials are used extensively [15,16]. The animal health workers involved in the project worked in Entebbe municipality either with the local government or as private practitioners. These health workers carry out diagnosis and treatment of animals mainly in the community. The CHWs in Busiro South HSD involved in the project not only treat childhood illnesses of diarrhoea, pneumonia, and malaria but also participate in educating the community on key public health issues, including AMR.

### 2.2. Project Team and Reciprocal Visits

This project was delivered as part of a 10-year international partnership between NTU, UK, and School of Public Health at Mak, Uganda. The partnership co-opted a multidisciplinary team for delivery of the project. This was essential due to the nature of the multifactorial challenges of AMR in humans, animals, and the environment. From the UK, specialist antimicrobial pharmacists and a medical microbiologist from BHT, a microbiology lecturer from School of Science and Technology, and an animal specialist from the School of Animal, Rural and Environmental Studies (ARES) at NTU took part in the interventions. In Uganda, project partners included: public health specialists, pharmacists including a clinical pharmacist, a veterinary doctor, and a microbiologist from Mak with support from health professionals from the Ministry of Health (MOH), ERRH, Wakiso district local government, and Entebbe Municipal Council. As part of the project, reciprocal visits between members from the UK and Uganda for planning, scoping, implementation, knowledge exchange, and sharing of best practices were held.

### 2.3. Project Planning and Stakeholder Engagement

The multidisciplinary project team conducted several meetings both physically and virtually before, during, and after implementation. The virtual meetings were held monthly using Skype and were attended by partners from both the UK and Uganda. The meetings facilitated project planning, implementation, monitoring, and evaluation as well as keeping track of the achievement of set goals. Other day-to-day communication to support implementation and timely completion of project tasks was achieved using a WhatsApp group. A google drive account was also set up for the team to access project documentation, such as previous meeting minutes, photos, and training materials. These communication avenues were invaluable for tracking progress of ongoing project activities and enhancing team work. Before and during project implementation, the project team held several meetings and engagements with various stakeholders in Uganda to ensure ownership, buy-in, and participation in planned activities. These stakeholders included government ministries (MOH and Ministry of Agriculture, Animal Industry and Fisheries—MAAIF), governmental parastatals (such as the National Drug Authority), professional associations (the Pharmaceutical Society of Uganda), training institutions (Mak), local governments (Wakiso district), health facilities (ERRH and lower level health facilities, such as health centre IIs, IIIs and IVs), local leaders (such as local council chairpersons), and the general community. The project team specifically engaged the MOH Technical Working Committee (TWC) on AMS, optimal access, and use, which is mandated to provide technical oversight of all AMS activities in the country. This engagement involved collaborative planning as well as regularly providing project updates in the TWC meetings and getting feedback that informed ongoing activities.

### 2.4. Enhancing Capacity of Health Practitioners, Community Health Workers, and School Pupils

The project held training workshops for HPs from both human and animal health to create awareness on AMR/AMS/IPC using a One Health approach. The workshops targeted selected HPs from government health facilities, including ERRH, as well as animal health workers in Wakiso district. The selection of HPs involved in the workshops was done in consultation with contacts at ERRH, Entebbe Municipal Council, and Wakiso District Health Office. Using the ‘training of trainers’ model, selected trained HPs were involved in training workshops for CHWs in AMR/AMS/IPC also using a One Health approach. The CHWs were from Kajjansi town council in Wakiso district where earlier NTU–Mak partnership interventions had been implemented [17]. All CHWs in the town council available at the time were involved in the workshops. In addition to the training of HPs and CHWs, the project also sensitised pupils in two primary schools in Wakiso district (St. Theresa and Kawotto Saviours Primary Schools) on AMR/AMS/IPC. St. Theresa’s is a government school in Entebbe municipality while Kawotto Saviours is a private school in Kajjansi town council, both in Wakiso district. These schools were selected in consultation with key stakeholders in Wakiso district.

### 2.5. Establishment of One Health Communities of Practice and University Student Engagement

The project set up two online CoPs involving individuals from human health, animal health, and the environment. The first CoP was for health professionals, including HPs, researchers, policy makers, and academics, while the second targeted undergraduate students of Mak from various disciplines, including environmental health, veterinary medicine, pharmacy, biomedical sciences, social sciences, and computer sciences. In addition to the CoPs, multidisciplinary students at Mak and microbiology students at NTU were involved in various activities to promote AMR/AMS/IPC, including seminars, webinars, and competitions. The competitions, which were to design AMS messages using innovative approaches, were held to commemorate WAAW 2019. The winners of the competition were recognized as a form of motivation to continue being antibiotic guardians in their respective settings.

### 2.6. Establishment of a Medicine and Therapeutics Committee

Working closely with the hospital pharmacist, the project supported the establishment of a MTC at ERRH. This included appointing a multi-disciplinary team, in accordance with the MOH MTC manual [18]. The aim of the MTC is to support the safe and effective use of medicines through evaluation of usage, and to develop guidelines and protocols for medicine prescription and administration, as well as other health commodity related activities at the hospital. The MTC at ERRH has 12 members and three sub-committees reflecting the main functions: supply chain; pharmacovigilance, and AMS. During the course of the project, the MTC held three meetings to elect members, establish roles and responsibilities, develop work plans including a procurement plan for the 2020/21 financial year, and define standard IPC practices at the hospital. The MTC was specifically involved in: selection of medicines to be used; monitoring and ensuring rational use of medicines as per the standard treatment guidelines; development of draft treatment protocols; as well as developing Standard Operating Procedures (SOPs) during the management of COVID-19 patients.

### 2.7. Project Evaluation

**Figure 1:**
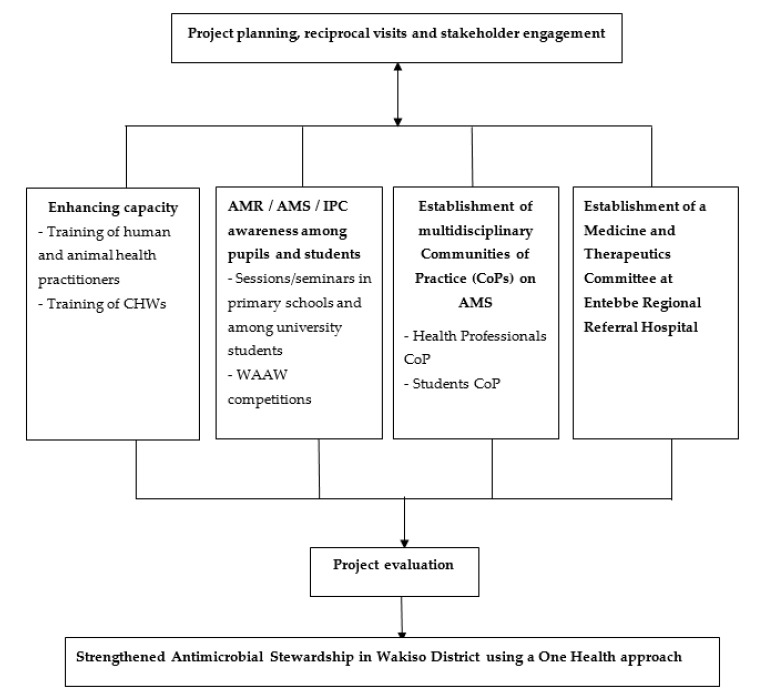
Summary of the project implementation.

The final project evaluation involved assessment of practices of the beneficiary HPs and CHWs, which was carried out three months after their respective training workshop. Specifically, the evaluation was aimed at establishing any changes in practices of the HPs and CHWs related to AMR/AMS/IPC following the workshops conducted as part of the project. The practices assessed among the HPs included: increased use of the Uganda Clinical Guidelines (UCG) when prescribing antimicrobials; increased diagnosis based on laboratory results; improved handwashing; and more patient guidance and counselling when they do not require antimicrobials. For the CHWs, practices, such as improved hand washing with soap as well as increased community sensitization on avoiding self-medication, consulting animal health professionals whenever animals were ill, and avoiding the use of human prescribed medicines in animals including poultry, were assessed. The evaluation among the HPs used a self-administered questionnaire while for the CHWs, a researcher-administered questionnaire was used. In addition, key informant interviews using telephone were held with selected key individuals from the various stakeholders involved in the project. The interviews included HPs and CHWs who participated in the workshops, particularly those with leadership roles, CoP members, Wakiso district health office staff, and facilitators of the training. In this paper, we present a summary of the key findings from the evaluation. The diagrammatic summary of the project implementation is shown in Figure 1.

## 3. Results

### 3.1. Reciprocal Visits

The project exchange visits lasted between one and two weeks depending on the timing of activities as well as the availability of the team members. The timing of these visits was crucial for the different teams to appreciate the level of, and approaches to AMS in the host country. The first UK team visit to Uganda in April 2019 was organised as a scoping visit. This was necessary to appreciate AMS activity in the country, including challenges, knowledge gaps, areas of sub-optimal antibiotic use, development needs at ERRH, and animal health AMS concerns to inform the project activities, including training of HPs and capacity support to ERRH. During this visit, several meetings were held with the ERRH administration, Wakiso district health and veterinary staff, MOH Department of Pharmaceuticals and Natural Medicines, and College of Veterinary Medicine, Animal Resources and Biosecurity at Mak. The second exchange visit was that of the ERRH pharmacist to the UK in June 2019. The pharmacist was involved in AMS discussions involving multi-disciplinary teams from BHT and NTU (City, Brackenhurst, and Clifton campuses). While at the Brackenhurst campus, which houses the School of Animal, Rural and Environment Sciences at NTU, the pharmacist learnt about the various animal projects there, including research on AMS. The pharmacist also spent some time in the microbiology department at the Clifton campus where he made a presentation to share experiences of AMS at ERRH and generally in Uganda among faculty. At BHT, the pharmacist spent time seeing AMS in practice on clinical ward rounds and in the pathology laboratory, and visited the medical microbiology laboratory where he gave a presentation to scientists. The pharmacist met with the medical director to discuss a potential memorandum of understanding with ERRH and how future work may be undertaken to support other collaborative activities. In the UK, the pharmacist was also involved in further project planning and work shadowing to learn more about AMS practices. The third exchange visit was of the UK team in September 2019 that involved a series of activities including: facilitation of three two-day AMS trainings among human and animal HPs; AMS seminars at Mak; and visits to primary schools to sensitize them on AMR and to launch an awareness competition. The final visit was by a Ugandan clinical pharmacist and lecturer at Mak in September 2020. This visit focused on AMS / AMR knowledge exchange, potential expansion of partnership work, discussions on final project activities including evaluation and dissemination, as well as exploring avenues for future collaboration between Uganda and the UK. In total, six UK members, including antimicrobial pharmacists, microbiologists, and an animal health specialist from NTU and BHT, travelled to Uganda over the two visits, while two Uganda pharmacists visited the UK as part of the project.

### 3.2. Training of Health Practitioners

In September 2019, the project team held training workshops for a multidisciplinary group of 86 health professionals from human and animal health on AMR/AMS/IPC. Among the trained HPs, 56 were from ERRH, 20 were from lower level health facilities in Wakiso district (Nsaggu HC II, Nakawuka HC III, Kajjansi HC IV, Zzinga HC II, Kitala HC II, Bussi HC II, and Kasanje HC III), and 10 were animal health professionals working within Entebbe municipality. Whereas all the human health HPs were selected from government health facilities, some of the animal health HPs were in private practice. The workshops for the HPs from both animal and human health were greatly appreciated by the participants as they shared experiences among themselves, and realised the similarities and close linkage between AMS among their professions. All HPs trained were involved in either prescription, administration, issuance of antimicrobials, or related clinical work. They included pharmacists, medical doctors, laboratory technicians, clinical officers, nurses, and veterinary practitioners. The two-day training workshops, held in Entebbe, were facilitated jointly by the UK and Uganda members of the project team. This ensured the local context of material and applicability was delivered during the training. The UK trainers included three antimicrobial pharmacists, two microbiologists, and an animal health specialist, while the Uganda team included three pharmacists, a veterinary doctor, and two environmental health scientists. The workshop sessions included: introduction to AMR/AMS/IPC; the World Health Organization (WHO) AMR competency framework for human health; prudent antibiotic use, actions, and barriers in human and animal health; use of UCG incuding how to use the Microguide app that hosts these guidelines provided by CwPAMS; the One Health approach; hand hygiene; and sharing of gained knowledge with others using the capability, opportunity, and motivation behaviour (COM-B) change model [19]. The workshop sessions were interactive and engaged participants in order to facilitate adult learning. Specifically, sessions included a pre- and post-assessment, group discussions, e-Bug AMR balloon experiment [20], hand washing demonstration using the Glow Germ Gel kit™ [21], case studies from both human and animal health, interactive games, sharing of past experiences, and a certificate awarding ceremony. From the pre- and post-training assessment, there was an improvement in the knowledge levels of the HPs after the trainings. Out of the 80 HPs who participated in the post assessment, 64 (80%) correctly defined AMR compared to 63.9% (53/83) who had done so during the pre-assessment. In the post-training evaluation, 73.8% (59/80) of the HPs stated that inadequate hand hygiene is one of the contributing factors of AMR compared to 21.7% (18/83) who did so during the pre-training assessment.

### 3.3. Training of Community Health Workers

A total of 227 CHWs from Kajjansi town council took part in training workshops conducted by a team of three HPs who were among those trained in the project as part of the ‘training of trainers’ model. The one-day workshop for CHWs, in groups of approximately 30, was conducted using interactive and engaging sessions similar to those used with HPs. Given that CHWs are involved in treatment of only three diseases (malaria, diarrhoea, and pneumonia) for infants less than five years under iCCM, and health promotion on key public health issues, their training was largely focused on creating awareness on AMR/AMS/IPC in their communities. The workshops included sessions on: introduction to antimicrobials and AMR; AMS at the community level; prevention of AMR in animals and humans; water, sanitation, and hygiene; food hygiene and safety; and IPC in communities. Similar to the workshops of HPs, a pre- and post-training assessment was undertaken for the CHWs. There was a notable improvement in knowledge of the CHWs in the post-training assessment in comparison with the pre-training survey. Out of the 212 CHWs who participated in the post-training assessment, 97.1% (206/212) reported that microorganisms can fail to respond to antimicrobials compared to 49.3% (111/225) in the pre-training assessment. In addition, 96.2% (204/212) of the CHWs reported in the post-training assessment that antimicrobials dumped in the environment can lead to AMR compared to 33.3% (75/225) in the pre-training assessment. Furthermore, 97.6% (204/212) of the CHWs agreed in the post-training assessment that inappropriate use of antimicrobials in livestock can lead to AMR compared to 36.4% (82/225) in the pre-training assessment. In the post-training assessment, all the CHWs (100%) felt that they were knowledgeable enough to educate their communities on preventing AMR through improved AMS and IPC following the training.

### 3.4. Increasing Awareness on AMS to Primary School Pupils and University Students

The project introduced and created awareness on AMR/AMS among over 300 pupils at St. Theresa and Kawotto Saviours primary schools in Wakiso district. These sessions were attended by pupils mainly in the upper classes of the schools (primary 5 to 7) as well as their teachers. These schools were twinned with two schools in the UK (Wingrave Church of England and Longwick Church of England primary schools). Pupils in both the Ugandan and UK schools also participated in a competition to develop AMR/AMS/IPC messages in commemoration of World Antibiotic Awareness Week (WAAW) 2019, and the winners received various awards. Winners in Uganda and the UK were selected for best poster / song / performance. Award ceremonies were held at the schools in Uganda and the UK to share the winning material among pupils, teachers, and parents to reinforce the messages and illustrate the importance of the global issue of AMR. Winners (four from Uganda and four from the UK) received an award of 25 GBP, a young antibiotic guardian t-shirt, and a certificate of appreciation for their participation. As part of the twinning of the Uganda and UK schools, ideas were shared on future collaboration, which will be explored in future. An initial activity carried out as part of the twinning was pupils from the Uganda schools writing pen pal letters to their UK counterparts and vice versa, which was well received by both groups and the administration of the schools. At Mak, the project organised three seminars and workshops on AMR / AMS /IPC, and interactions with NTU students. The seminars were attended by over 120 students from the UK and Uganda from different programs, including environmental health, pharmacy, microbiology, and environment. The project also launched an AMR awareness competition among Makerere University Environmental Health Students’ Association (MUEHSA) and NTU students for them to design appropriate messages for community sensitization. During the World Antibiotic Awareness Week (WAAW) in November 2019, the project held an award giving ceremony in which the four winners of the competition from Mak and NTU each received a cash prize of 25 GBP as well as a certificate of appreciation.

### 3.5. Establishment of One Health AMS Communities of Practice

Through stakeholder engagement at initiation of the project, the need for two online CoPs, one for health professionals and the other for students (as opposed to the earlier planned one), was identified and established. The aim of the CoPs was to provide a platform for sharing resources, opportunities, and materials on AMR, AMS, and IPC targeting both human and animal health, as well as enhance sustainability as these would continue after the project duration. The *Antimicrobial Stewardship, Optimum Access and Use in Uganda* CoP for health professionals is hosted by the MOH TWC on AMS, optimum access, and use. The *Students for Antimicrobial Stewardship* CoP is a Facebook group that was formed after the realisation of the need for students to work together, in a multidisciplinary setting, at an early stage in their careers to tackle AMR. This platform is managed by five AMR champions from the following schools at Mak: veterinary medicine; biosecurity; biotechnical, and laboratory science; health sciences; and public health. Currently, the students’ CoP has 162 members while the one for health professionals has 321 members, with membership of both groups steadily increasing. The health professionals’ CoP has sent out over 50 emails with resources, opportunities, and other materials concerning AMR/AMS from Uganda, the UK, and globally. One example of how this has changed engagement with this work is that some members of the health professionals CoP have submitted abstracts to conferences, which they learnt about from the online platform. The students CoP has sent over 20 messages on opportunities for students to participate in. These opportunities have included attending webinars and conferences, such as the 4th National Conference on AMR held in Kampala, Uganda in 2019. In addition, students on their CoP have appreciated the importance of working in multi-disciplinary teams to tackle AMR, which they are likely to utilize during their future professional work.

### 3.6. Evaluation of the Trained HPs and CHWs

From the project evaluation, there was a positive change in practices among the HPs and CHWs following the training. Out of the 77 HPs who participated in the evaluation, 68 (88.3%) stated that they had adopted new practices from the project training. Out of the 68 HPs who adopted new practices, 39 (57.3%) reported improved handwashing, over half 36 (52.9%) reported an increase in use of the UCG when prescribing antimicrobials, and 35 (51.5%) reported a reduction in the quantity of unnecessary antibiotics given per patient. Among the 77 HPs, 48 (62.3%) reported having faced challenges when attempting to become an antimicrobial steward in their setting. These challenges included stock out of drugs, 29 (60.4%); lack of personal protective equipment (PPE), including gloves and masks, 19 (39.6%); and insufficient laboratory capacity, 17 (27.1%) (Table 1).

From the qualitative evaluation of the project, the HPs reported that they were using the UCG more during the prescribing of antimicrobials, and they had also reduced the quantities of antibiotics given per patient when appropriate to do so. Improved prescription practices among the HPs also led to improved availability of antimicrobials at the various health facilities as demonstrated in the quotation below.

*“At the facility nowadays, I only give out amoxicillin where necessary as per the guidelines. I do not just give out antibiotics anymore. For this reason, I am now able to save amoxicillin tablets for patients who really need them, and I also give out the medication in the right dose. This has helped me reduce on the number of times I go to look for medicines from other facilities due to reduced stock-outs at my facility.”*

Health worker, Bussi health centre II

Among the 226 CHWs who participated in the evaluation, 204 (90.3%) reported improved practices attributable to the training. The majority, 183 (81%), of the CHWs reported increased handwashing with soap, 175 (77.4%) had encouraged community members to improve personal hygiene and general sanitation, 151 (66.8%) had encouraged community members to take the full dose of their prescribed medication, while 130 (57.2%) had encouraged farmers to always consult veterinary professionals whenever their animals were ill. Following the training, 69 CHWs (30.5%) reported having reached between 50 to 100 community members and health educated them on AMR, while 27 (12%) reached over 100 community members (Table 2).

Qualitative evaluation among the CHWs confirmed improved practices amongst them regarding the prevention of AMR in the community, particularly regarding animal husbandry, such as observing antibiotic withdrawal periods among animals before consuming their products, not eating deceased animals that were recently on treatment, reduction of self-prescription for animals, and reduction in use of human-prescribed antimicrobials among animals as mentioned in the quotations below.

*“Before the training, I was among the people who used to slaughter sick chicken which were under treatment or those that had died while receiving medication. During the training, I learnt that we should never slaughter sick animals undergoing treatment. I now also know that to slaughter a chicken recently on treatment for consumption, you must wait for 7 or more days so that the medication is no longer present in its body hence not consuming small doses of the drug from the chicken which can contribute to AMR.”*

Female CHW, Kajjansi Town Council

*“For us, we thought that medicine that has been prescribed for humans could be used the way we wanted to treat animals especially antiretroviral drugs which we used to give pigs, and amoxicillin capsules to chicken. However, at the training I learnt that it is very dangerous to give human medicine to animals, and we should always call the veterinary doctor to treat our animals and prescribe the medicine for them, other than treating the animals ourselves.”*

Male CHW, Kajjansi Town Council

## 4. Discussion

Improper use of antimicrobials including non-compliance with guidelines is a contributing factor to AMR [4], hence the need for interventions targeting improvement of access to and appropriate use of antimicrobials. There is limited data on AMS programs in Africa, including Uganda [7,22], hence our project provides an important contribution to the existing body of knowledge on how to address the growing burden of AMR. The need for a One Health approach to promote AMS is well established internationally [5], and is translated into the local Ugandan context through the NAP [2]. The One Health approach used in the project was made possible through the multidisciplinary nature of our team with expertise in human and animal health, and environmental health. The strength of this team was fostered through reciprocal relationships and engagement with a variety of in-country stakeholders, including policy makers, such as the MOH and MAAIF. The project was delivered through multiple parallel interventions, including the training of HPs and CHWs, the creation of AMS awareness among university students and school pupils, as well as the establishment of CoPs and a hospital MTC. Our project is one of the few that have delivered multiple interventions at both health facility and community levels using a One Health approach in Uganda so provides a good contribution to the NAP in the fight against AMR.

In line with WHO recommendations, our training approach focused on both human and animal HPs [9]. In addition, the training of HPs on AMR/AMS/IPC took a One Health approach where an emphasis was given to the use of antimicrobials in humans and animals, and their link to the environment. This was necessary to truly embed the principles of One Health into the training component of our project. Indeed, with a multidisciplinary team from Uganda and the UK facilitating the training, the goal of ensuring One Health was achieved. The project was able to translate AMS principles across various primary health care levels in Uganda as the training involved HPs from ERRH and lower level health facilities. Whereas these lower level health facilities in Uganda (health centre IIs, IIIs and IVs) are often ignored for AMS and other interventions, they provide health care to a good portion of the population, particularly in rural areas that have limited access to hospitals [23]. The use of UK trainers in addition to those from Uganda was of benefit for both countries. These trainers brought their unique expertise around infection prevention and the UK’s principles of AMR and AMS practice. On the other hand, the UK trainers were exposed to the issues of AMS and AMR in a developing country context, and community-based approaches of tackling global health challenges, particularly AMR. The project also facilitated bi-directional learning between the two countries, which can inform other health partnerships. The use of participatory training techniques, such as demonstrating handwashing using the Glow Germ Gel Kits, facilitated learning and knowledge retention as opposed to traditional didactic methods.

In addition to the training of HPs, our project trained CHWs on AMR/AMS/IPC using the ‘training of trainers’ model. AMS programs are largely hospital based and have predominantly focused on HPs, such as laboratory scientists, nurses, clinicians, pharmacists, and microbiologists [24]. Our study went beyond this and involved CHWs as they are key to health service delivery at the community level in Uganda as well as other developing countries. The CHWs trained in our project have a primary responsibility of health education and promotion in their communities while some had an extra role on iCCM. From the evaluation results, it was evident that training of the CHWs enhanced their capacity to contribute to AMR through sensitization of the general population on the appropriate use of antibiotics, particularly in humans, but also in animals among other related issues. Miscommunication at the community level has been identified as a major challenge to addressing AMR in Africa [25], hence CHWs are a critical part of the health workforce to contribute to the NAP in the local setting. Given the training of CHWs had a component of IPC, they were subsequently invaluable in the promotion of sanitation and hygiene during the emergency public health measures undertaken in response to the COVID-19 pandemic in the communities. The use of the ‘training of trainers’ model, where the trained HPs later trained CHWs, is an indication of the health partnership’s commitment towards building local capabilities in primary health care delivery. In addition, using trained practitioners to train another health cadre enhances health promotion and ensures cultural appropriateness [26].

Awareness of basic hygiene principles and infection control are at the core of AMR education [5]. The AMS activities we implemented in schools will help to promote intergenerational awareness on AMR, as well as facilitate pupils becoming antimicrobial guardians in the future. Such pupils are also likely to promote proper AMS and IPC practices, such as handwashing with soap, at critical times among peers and family members. Our project also involved undergraduate students from various disciplines concerning human and animal health from Uganda and the UK in AMR awareness-raising activities, including seminars, webinars, and competitions. Although these students may have earlier been exposed to AMR as part of their studies, it cannot be guaranteed that they had adequate knowledge and skills on AMS, which would be reflected in their practice. At the undergraduate level, many prescribers and students of other professions may not be confident in their preparedness to deal with AMS. For instance, a survey among fourth-year medical students in the United States revealed that only one-third of those surveyed considered themselves as being adequately prepared in basic antimicrobial use [27]. Similarly, a study among paramedical students in Ethiopia revealed that less than half of the students surveyed had adequate knowledge of AMR [28]. These gaps in knowledge among university students reflect the need for AMS awareness initiatives to be incorporated into educational training curricula. Involving pupils and students in AMS / IPC interventions is therefore important as they are the future generation, and it also contributes to ensuring the sustainability of project activities.

Our project facilitated the establishment of an MTC at ERRH to support appropriate prescribing of antimicrobials and related activities. Having an MTC at a hospital is one of the recommended steps in setting up an AMS program [29], hence it is an important contribution to the NAP. During the COVID-19 pandemic, ERRH was the first facility to handle cases with disease in the country, and the rapid and comprehensive support from the MTC to guide the hospital response was timely. The current existence and operation of the MTC at the hospital is a key achievement of our project given that it continues to support day-to-day operations at the facility, hence contributing to the sustainability of our project interventions. Establishment of the CoPs on AMS was also instrumental in ensuring sustainability as they continue to enhance knowledge exchange among human and animal health professionals and students. The CoPs continue to be integral to building awareness and sharing best practices on AMR/AMS/IPC. Given the COPs are online, the UK project team are able to stay engaged and be involved when not in Uganda. Indeed, online CoPs have been demonstrated to have a wider reach and keep participants engaged in comparison with physical ones, particularly in this digital age [30,31]. Having set-up the health professionals’ CoP as part of the MOH TWC on AMS, optimum access and use will also contribute towards its sustainability beyond the project duration.

The pre- and post-training assessment and project evaluation revealed an improvement in HPs’ and CHWs’ knowledge of AMR and their practices to promote AMS and IPC. Specifically, the evaluation results showed that the training of HPs substantially improved the organizational culture for the majority, with 88.3% adopting new practices around AMR/AMS/IPC in line with the national requirement as prescribed in the UCG. Studies in different parts of Africa, such as South Africa [32], have also recorded change in organizational culture following the implementation of an AMS program. This change in practice among HPs involved in our study resulted in improved availability of antimicrobials as prescriptions and use were optimized. However, the evaluation revealed that health systems challenges, such as stock out of medicine, inadequate human resources, and lack of PPE for hospital staff, could impede AMS promotion if not equally tackled. In the case of CHWs, the evaluation results demonstrated how training them has a wider impact on improving positive health outcomes across communities, with many CHWs having sensitized between 50 and 100 community members on AMR/AMS within three months following the training. In addition, training the CHWs was instrumental in promoting the One Health approach, which was evident through encouraging livestock farmers to consult with a veterinary specialist regarding the health of their animals in the community. Animal husbandry is an important practice in the project community [15] and the East African region in general [25] so it is critical it is considered while implementing AMS interventions. This practice is reported to bring with it a high burden of what Ampaire et al. [25] referred to as “community-acquired infections”. There is a high rate of antibiotics misuse and poor engagement with veterinary professionals among livestock farmers at the community level in Africa [33]. Therefore, the ability of CHWs to support improved practices regarding management of animal conditions should be integrated in future AMS activities to contribute to the fight against AMR in Uganda and beyond.

One of the strengths of our project is that it was implemented as part of a 10-year established health partnership between NTU and Mak with existing structures, intellectual capital, as well as local and global resources and networks that will contribute to strengthening the primary health care system in Uganda. In addition, our project embraced the One Health approach and targeted AMS interventions at both the health facility and community levels as well as including primary schools and university students, which is worth mentioning. A limitation of our project was its limited scope given it involved one hospital, a few lower level health facilities, two primary schools, and selected university students. The involvement of animal health workers in project activities was also low compared to those from human health, which can be improved in the future. Nevertheless, being a small pilot project, the achievements and lessons learnt will be instrumental in informing our future partnership activities to strengthen AMS in Uganda and further contribute to the NAP.

## 5. Conclusions

Adoption of a One Health approach in our project facilitated multidisciplinary efforts, including training human and animal HPs, to increase awareness and contribute towards improving AMS at health facilities and in the community. Reciprocal visits and establishment of CoPs fostered bi-directional learning and knowledge transfer on AMS between the UK and Uganda. The achievements of this project can inform the design of large-scale AMS interventions in support of implementation of the Uganda AMR National Action Plan.

## Figures and Tables

**Table 1:** Evaluation results of health practitioners.

	Frequency (N = 77)	Percentage (%)
**Gender**		
Male	29	37.7
Female	48	62.23
**Nature of practitioner**		
Human health worker	72	93.5
Animal health worker	5	6.5
**Found the training helpful in their day-to-day activities**		
Yes	71	92.2
No	6	7.8
**Presence of new practices adopted after training**		
Yes	68	88.3
No	6	7.8
Not sure	3	3.9
* Adopted new practices after training (n = 68)		
Increased use of UCG when prescribing antimicrobials	36	52.9
Diagnosis based on laboratory results	30	44.1
Sending more samples to the laboratory	24	35.3
Improved handwashing	39	57.3
Monitoring of my prescribing patterns	26	38.2
Patient guidance and counselling when they do not need antibiotics	31	45.6
Reduction of the number of antibiotics given per patient	35	51.5
Reduction of the use of injectables at out-patient department	19	27.9
** Others	32	47.1
**Were using the CwPAMS microguide app**		
Yes	33	43.4
No	39	50.0
Not applicable because they are Veterinary officers	5	6.6
**Faced challenges when attempting to become antimicrobial stewards**		
Yes	48	62.3
No	29	37.7
**Challenges faced (n = 48)**		
Drug stock outs	29	60.4
Lack of prescribing materials/guidelines	11	22.9
Lack of hand washing facilities and/or supplies	11	22.9
Lack of gloves, masks and/or other PPE	19	39.6
Insufficient laboratory capacity	17	27.1
Lack of first line antibiotics	13	16.9
Supervisors not very supportive in AMS matters	5	10.4
*** Others	22	45.8

* Multiple response question; ** Other practices included advising against self-medication, encouraging use of organic feeds for poultry and livestock, health education on adherence to drugs, waste segregation, and training other HPs who did not attend the training; *** Other challenges included poor attitude of fellow HPs, patients demanding for antibiotics, self-medication by patients, and lack of flexibility among some prescribers.

**Table 2:** Evaluation results of community health workers.

Gender	Frequency (N = 226)	Percentage (%)
Male	47	20.8
Female	179	79.2
**Nature of CHW**		
Involved in iCCM	111	49.1
Not involved in iCCM	115	50.9
**Change in practice after the training**		
Yes	204	90.3
No	22	9.7
* New practices adopted individually after the training (n = 204)		
Increased use of treatment guidelines for childhood illness	90	39.8
Patient guidance/counselling when they do not need antibiotics	74	32.7
Proper disposal of medical waste and medicine	111	49.1
Encouraged community members to take full dose of medication	151	66.8
Encouraged community members to stop self-medication	159	70.4
Encouraged community members to follow doctors’ prescriptions	144	63.7
Increased hand washing with soap	183	81.0
Encouraged community members to improve personal hygiene and general sanitation	175	77.4
Encouraged food hygiene and safety	135	59.7
Promoted the safe water chain	172	76.1
Encouraged farmers to consult veterinary doctors whenever animals were ill	130	57.2
Encouraged farmers not to use human medicines in poultry and animals	112	49.6
** Others	69	30.5
Number of people sensitized about AMR in the community		
<50	130	57.5
50–100	69	30.5
>100	27	12.0
**Faced challenges when attempting to become antimicrobial guardians in the community**		
Yes	208	92.0
No	18	8.0
**Challenges faced when attempting to become antimicrobial guardians (n = 208)**		
People do not understand AMR	132	62.9
Needed more training	136	64.8
AMR is complicated	44	21.0
*** Others	61	29.1

* Multiple response question. ** Others included advising against sharing of drugs, proper drug storage, advising against eating of dead animals, and burying dead animals. *** Other challenges included poor attitude of community members, poverty, and disrespect for CHWs.
